# A Nomogram Model to Predict Post-Progression Survival in Esophageal Squamous Cell Carcinoma Patients With Recurrence After Radical Resection

**DOI:** 10.3389/fonc.2022.925685

**Published:** 2022-07-07

**Authors:** Changsen Leng, Yingying Cui, Junying Chen, Kexi Wang, Hong Yang, Jing Wen, Jianhua Fu, Qianwen Liu

**Affiliations:** ^1^ State Key Laboratory of Oncology in South China, Collaborative Innovation Center for Cancer Medicine, Sun Yat-sen University Cancer Center, Guangzhou, China; ^2^ Department of Thoracic Surgery, Sun Yat-sen University Cancer Center, Guangzhou, China; ^3^ Guangdong Esophageal Cancer Institute, Guangzhou, China; ^4^ Department of Hematologic Oncology, Sun Yat-sen University Cancer Center, Guangzhou, China; ^5^ Department of Thoracic Surgery, Sun Yat-sen Memorial Hospital, Sun Yat-sen University, Guangzhou, China

**Keywords:** esophageal squamous cell carcinoma (ESCC), recurrence, post-progression survival, nomogram, prognostic model

## Abstract

**Background:**

Esophageal squamous cell carcinoma (ESCC) is characterized clinically by frequent recurrence, leading to a poor prognosis after radical surgery. The aim of this study was to identify a prognostic nomogram to predict the post-progression survival (PPS) of ESCC patients based on the features of primary tumor and recurrence.

**Methods:**

A total of 234 ESCC patients who underwent recurrence after radical surgery were enrolled in this study. The independent prognostic factors screened by the univariate and multivariate Cox regression analysis were subsequently used to construct a nomogram. The predictive performance of the nomogram was evaluated with the concordance index (C-index), decision curve, and the area under the receiver operating characteristic curve (AUC) and validated in two validation cohorts. The Kaplan-Meier curves of different recurrence patterns were analyzed.

**Results:**

The prognostic nomogram of PPS was established by integrating independent prognostic factors, including age, body mass index, number of lymph node dissection, recurrence pattern, and recurrence treatment. The nomogram demonstrated good performance, with C-index values of 0.756, 0.817, and 0.730 for the training and two validation cohorts. The 1-year AUC values were 0.773, 0.798, and 0.735 and 3-year AUC values were 0.832, 0.871, and 0.791, respectively. Furthermore, we found that patients with bone metastasis displayed the worst PPS compared to other isolated recurrence patterns.

**Conclusion:**

We constructed a nomogram to reliably predict PPS, which would be valuable to provide individual managements for ESCC patients after radical surgery.

## Introduction

Esophageal cancer (EC) ranks seventh in terms of global cancer incidence, leading to more than 540,000 deaths annually (1). EC is characterized by an unfavorable prognosis with a general 5-year overall survival (OS) rate of less than 10% (2). With the improvement of surgical techniques, the 3-year OS of nonmetastatic EC after esophagectomy has been improved to around 70% in recent years (3, 4). However, local recurrence and/or distant metastasis happened to more than 50% of EC patients within 3 years after esophagectomy (5, 6). The prognosis for recurrent EC patients after surgery is poor, and the median survival time is only 3-10 months (6). Therefore, it is urgent to find the influencing factors related to the post-progression survival (PPS) of recurrent EC after surgery.

Currently, several models have been showed to predict postoperative OS and progression-free survival (PFS) for EC by using primary tumor characteristics (7, 8). Whereas, compared to OS and PFS, the survival after relapse of EC patients may be influenced by characteristics of the primary tumor as well as the recurrence-related features, such as recurrence patterns, metastatic sites, and recurrence interval (9, 10). According to a study that investigated 193 esophageal squamous cell carcinoma (ESCC) patients with recurrence, both primary pathological TNM stage and treatment regimen after recurrence were independent prognostic factors for PPS (11). However, Su *et al.* have reported that only the recurrent information, including the time of recurrence, pattern of recurrence, and treatment after recurrence were independent prognostic factors for ESCC patients with recurrence after radical esophagectomy (5). Furthermore, Parry *et al.* confirmed that the pattern of recurrence and the number of recurrent locations were independent prognostic factors for PPS (6). Butter *et al.* showed that resection margin status of the primary tumor, the number of positive lymph nodes after neoadjuvant therapy, age at recurrence, and location of recurrence were associated with PPS (12). These previous PPS-related findings, however, were based on univariate/multivariate analysis which could not accurately exhibit their proportion of contribution to the survival of EC patients with recurrence (5, 11, 13, 14). Hence, it is essential to establish a prognostic predictive system to better evaluate the PPS of individual EC patients. However, as far as we know, no relevant studies have been carried out.

ESCC is the main pathological type of EC globally (15). This study aims to integrate the primary and recurrent tumor characteristics and develop a prognostic nomogram to predict the PPS of recurrent ESCC patients after radical resection and evaluate its prognostic predictive performance.

## Materials and Methods

### Study Population and Inclusion Criteria

Primary ESCC patients with recurrence after radical resection at the Department of Thoracic Surgery, Sun Yat-sen University Cancer Center from 1999 to 2016 were enrolled in this study. The key eligibility criteria included: (1) patients underwent McKeown or Ivor-Lewis esophagectomy; (2) histologically confirmed ESCC; (3) R0 resection (16); (4) initially stage IB-IVA (in accordance with the 8th Edition of the American Joint Committee on Cancer (AJCC) Staging system) (17); (5) patients with comprehensive clinical data; (6) at least 10 months of follow-up. Patients were excluded if: (1) patients had a secondary malignancy; (2) patients received neoadjuvant chemotherapy and/or radiotherapy. All eligible ESCC patients were enrolled in the training cohort. Patients were then randomly allocated in a 1:1 ratio to two validation cohorts. This study was approved by the Institutional Review Board of Sun Yat-Sen University Cancer Center with an approval batch number of SL-B2022-248-02 and was performed in line with the ethical guidelines of the 2013 Declaration of Helsinki.

### Data Collection

Demographic data involving age, gender, smoking, drinking, diet, weight loss (since onset), body mass index (BMI), previous history of diabetes or hypertension, diagnostic date, and family history of tumor was incorporated into this study. Pathologic characteristics, including histological type, tumor location (upper third and middle/lower third segment of the esophagus), tumor size, tumor differentiation (well/moderately/poorly), number of lymph node dissection (LND), the number of positive lymph nodes, T-stage, N-stage, and AJCC stage, were evaluated by at least two pathologists specialized in tumor pathology. Tumor size was measured as the largest dimension of the lesion. The ratio of the number of positive lymph nodes to LND was defined as the lymph node metastasis ratio (LNR). Information of intraoperative thoracic duct ligation and postoperative anastomotic leakage was collected as well. Treatments after esophagectomy surgery were consisted of chemotherapy, radiotherapy, chemoradiotherapy, and no treatment. Recurrence treatment referred to the treatment after tumor recurrence, which included chemotherapy, radiotherapy, chemoradiotherapy, surgery, targeted therapy and/or immunotherapy, and supportive care.

Regular follow-up was carried out every 3-6 months after surgery. Recurrent information of ESCC patients containing the time to recurrence, site of recurrence, and the recurrent pattern was determined by endoscopic and/or imaging examinations, such as contrast-enhanced CT, MRI, bone scintigraphy, ultrasonography, and/or PET/CT. The biopsy was performed if those exams cannot clearly define the recurrence. The time to recurrence was defined as the time from the radical surgery to the first recurrence.

Recurrence patterns were categorized as local-regional recurrence and distant metastasis. Local-regional recurrence was defined as the recurrence in the following areas: the anastomotic stoma and the lymph nodes in the mediastinal, upper abdominal, and cervical areas. The terms “anastomotic only, cervical only, mediastinal only, and abdominal only” referred to isolated recurrence in anastomotic stoma and the lymph nodes in the mediastinal, upper abdominal, and cervical areas. “Multiple local” was defined as the recurrence in multiple local-regional areas. Distant metastasis was defined as any spread of disease beyond the local-regional recurrence. The terms “lung only, liver only, bone only, and pleura only” referred to isolated recurrence in lung, liver, bone, and pleura. The term “other distant only” referred to isolated distant metastasis in other less common areas. “Multiple distant” was defined as the recurrence in multiple areas including distant metastases. Local-distant recurrence referred to the simultaneously observed local recurrence as well as distant metastasis, which was also classified in the distant metastasis group.

### Statistical Analysis

In the present study, PPS was the interested endpoint and was defined as the time from the occurrence of tumor progression to the date of death or last follow-up. The Kaplan-Meier method was applied to estimate the survival of ESCC patients after disease recurrence and the survival differences were compared with a log-rank test. The optimal cutoff value for continuous variables was calculated by X-tile (Yale University, Version 3.6.1) (18). After checking the proportional hazards assumption with graphical method by using SPSS (19), the univariate Cox regression analysis was employed to select the clinicopathological and recurrent features by using “survival” and “survminer” packages in R. Next, variables were subjected to the least absolute shrinkage and selection operator (LASSO) analysis by using the “glmnet” package in R to identify better predictors. The multivariate Cox regression analysis was performed to further screen independent prognostic factors in ESCC patients. Then, a nomogram integrating these prognostic factors was constructed by using the “rms” package to predict the PPS. These prognostic factors were assigned points in proportion to their contribution to PPS. To verify its predictive accuracy, split-sample validation and the 1000 bootstrap resamples were applied for internal validation in this study (20). Calibration curves were used to assess the consistency between nomogram prediction and the actual outcome (20). The predictive performance of the nomogram was evaluated by the receiver operating characteristic (ROC) curve, the area under the ROC curve (AUC), and the concordance index (C-index) (20, 21). The clinical utility of the nomogram was evaluated with the decision curve analysis (22). References of R-packages were listed in **Supplementary Table 1**.

Clinicopathological characteristics differences were compared between two validation cohorts with chi-square or Fisher’s exact test according to sample size. *P*-values < 0.05 were considered statistically significant. All statistical analyses were conducted using R (Version 4.0.3, http://www.r-project.org) and SPSS (Version 23, IBM, New York, USA).

## Results

### Patient Clinicopathologic Characteristics

A total of 234 eligible ESCC patients who underwent recurrence after radical esophagectomy were enrolled in this study, with a median follow-up of 47.5 months (range 10.3-154.5 months). The latest follow-up time was 31^st^, December 2021 and 110 patients died during the follow-up period. The average age of patients was 57 years (range 32-75 years), and the majority of patients were male (n = 189, 80.8%). Among them, 191 (81.6%) patients were initially diagnosed with squamous cell carcinoma in the middle or lower third of the esophagus. The median time to recurrence was 12.5 months. Of all recurrent patients, 91 (38.9%) experienced distant metastasis and 143 (61.1%) had only local-regional recurrence. Median PPS of patients with distant metastasis and local-regional recurrence were 16.9 and 27.1 months, respectively. The 1- and 3-year PPS rates of ESCC patients were 69.0% and 34.9%, respectively. The specific clinicopathologic and recurrent characteristics of patients in the training cohort (n = 234) and two validation cohorts (n = 117) are listed in **Table 1**. There were no significant differences between the two validation cohorts regarding the general characteristics of patients.

**Table 1:** Clinical and pathologic characteristics of ESCC patients.

Characteristics	Levels	Training cohort(n=234)	Validation cohort 1(n=117)	Validation cohort 2(n=117)	*P*-value
Age	≤65 years	202 (86.3%)	99 (84.6%)	103 (88.0%)	0.568
	>65 years	32 (13.7%)	18 (15.4%)	14 (12.0%)	
Gender	Male	189 (80.8%)	98 (83.8%)	91 (77.8%)	0.320
	Female	45 (19.2%)	19 (16.2%)	26 (22.2%)	
Tumor location	Upper third	43 (18.4%)	21 (17.9%)	22 (18.8%)	1.000
	Middle or lower third	191 (81.6%)	96 (82.1%)	95 (81.2%)	
Hypertension	No	213 (91.0%)	105 (89.7%)	108 (92.3%)	0.647
	Yes	21 (9.0%)	12 (10.3%)	9 (7.7%)	
Diabetes	No	227 (97.0%)	115 (98.3%)	112 (95.7%)	0.446
	Yes	7 (3.0%)	2 (1.7%)	5 (4.3%)	
Smoking	No	88 (37.6%)	42 (35.9%)	46 (39.3%)	0.686
	Yes	146 (62.4%)	75 (64.1%)	71 (60.7%)	
Drinking	No	149 (63.7%)	72 (61.5%)	77 (65.8%)	0.587
	Yes	85 (36.3%)	45 (38.5%)	40 (34.2%)	
Family tumor history	No	192 (82.1%)	99 (84.6%)	93 (79.5%)	0.394
	Yes	42 (17.9%)	18 (15.4%)	24 (20.5%)	
Diet	Normal	88 (37.6%)	46 (39.3%)	42 (35.9%)	0.716
	Semi-liquid	126 (53.8%)	60 (51.3%)	66 (56.4%)	
	Fluids	20 (8.5%)	11 (9.4%)	9 (7.7%)	
Weight loss	≤2.5 kg	176 (75.2%)	87 (74.4%)	89 (76.1%)	0.880
	>2.5 kg	58 (24.8%)	30 (25.6%)	28 (23.9%)	
BMI	≤18.8 kg/m^2^	40 (17.1%)	24 (20.5%)	16 (13.7%)	0.224
	>18.8 kg/m^2^	194 (82.9%)	93 (79.5%)	101 (86.3%)	
Diagnostic date	Before 2010	94 (40.2%)	47 (40.2%)	47 (40.2%)	1.000
	After 2010	140 (59.8%)	70 (59.8%)	70 (59.8%)	
Thoracic duct ligation	No	116 (49.6%)	57 (48.7%)	59 (50.4%)	0.896
	Yes	118 (50.4%)	60 (51.3%)	58 (49.6%)	
Anastomotic leakage	No	199 (85.0%)	103 (88.0%)	96 (82.1%)	0.271
	Yes	35 (15.0%)	14 (12.0%)	21 (17.9%)	
Differentiation	Well	49 (20.9%)	27 (23.1%)	22 (18.8%)	0.130
	Moderately	130 (55.6%)	69 (59.0%)	61 (52.1%)	
	Poorly	55 (23.5%)	21 (17.9%)	34 (29.1%)	
T stage	T1b	13 (5.6%)	6 (5.1%)	7 (6.0%)	0.182
	T2	39 (16.7%)	14 (12.0%)	25 (21.4%)	
	T3	178 (76.1%)	94 (80.3%)	84 (71.8%)	
	T4a	2 (0.9%)	1 (0.9%)	1 (0.9%)	
	T4b	2 (0.9%)	2 (1.7%)	0 (0%)	
N stage	N0	75 (32.1%)	33 (28.2%)	42 (35.9%)	0.502
	N1	88 (37.6%)	48 (41.0%)	40 (34.2%)	
	N2	52 (22.2%)	25 (21.4%)	27 (23.1%)	
	N3	19 (8.1%)	11 (9.4%)	8 (6.8%)	
AJCC stage	IB	8 (3.4%)	3 (2.6%)	5 (4.3%)	0.374
	IIA	31 (13.2%)	12 (10.3%)	19 (16.2%)	
	IIB	42 (17.9%)	22 (18.8%)	20 (17.1%)	
	IIIA	15 (6.4%)	5 (4.3%)	10 (8.5%)	
	IIIB	117 (50.0%)	62 (53.0%)	55 (47.0%)	
	IVA	21 (9.0%)	13 (11.1%)	8 (6.8%)	
Tumor size	≤2.0 cm	32 (13.7%)	13 (11.1%)	19 (16.2%)	0.341
	>2.0 cm	202 (86.3%)	104 (88.9%)	98 (83.8%)	
Number of LND	≤10	28 (12.0%)	13 (11.1%)	15 (12.8%)	0.647
	>10, ≤18	58 (24.8%)	32 (27.4%)	26 (22.2%)	
	>18	148 (63.2%)	72 (61.5%)	76 (65.0%)	
LNR	≤0.07	131 (56.0%)	63 (53.8%)	68 (58.1%)	0.598
	>0.07	103 (44.0%)	54 (46.2%)	49 (41.9%)	
Treatment after surgery	No	164 (70.1%)	78 (66.7%)	86 (73.5%)	0.325
	CT	53 (22.6%)	31 (26.5%)	22 (18.8%)	
	RT	6 (2.6%)	4 (3.4%)	2 (1.7%)	
	CRT	11 (4.7%)	4 (3.4%)	7 (6.0%)	
Time to recurrence	≤6.2 months	40 (17.1%)	25 (21.4%)	15 (12.8%)	0.118
	>6.2 months	194 (82.9%)	92 (78.6%)	102 (87.2%)	
Recurrence pattern	Local-regional	143 (61.1%)	76 (65.0%)	67 (57.3%)	0.283
	Distant metastasis	91 (38.9%)	41 (35.0%)	50 (42.7%)	
Lung only	No	215 (91.9%)	106 (90.6%)	109 (93.2%)	0.632
	Yes	19 (8.1%)	11 (9.4%)	8 (6.8%)	
Liver only	No	222 (94.9%)	112 (95.7%)	110 (94.0%)	0.767
	Yes	12 (5.1%)	5 (4.3%)	7 (6.0%)	
Bone only	No	225 (96.2%)	114 (97.4%)	111 (94.9%)	0.499
	Yes	9 (3.8%)	3 (2.6%)	6 (5.1%)	
Pleura only	No	230 (98.3%)	116 (99.1%)	114 (97.4%)	0.622
	Yes	4 (1.7%)	1 (0.9%)	3 (2.6%)	
Other distant only	No	224 (95.7%)	112 (95.7%)	112 (95.7%)	1.000
	Yes	10 (4.3%)	5 (4.3%)	5 (4.3%)	
Multiple distant	No	225 (96.2%)	113 (96.6%)	112 (95.7%)	1.000
	Yes	9 (3.8%)	4 (3.4%)	5 (4.3%)	
Local Distant	No	206 (88.0%)	105 (89.7%)	101 (86.3%)	0.546
	Yes	28 (12.0%)	12 (10.3%)	16 (13.7%)	
Anastomotic only	No	223 (95.3%)	112 (95.7%)	111 (94.9%)	1.000
	Yes	11 (4.7%)	5 (4.3%)	6 (5.1%)	
Cervical only	No	177 (75.6%)	89 (76.1%)	88 (75.2%)	1.000
	Yes	57 (24.4%)	28 (23.9%)	29 (24.8%)	
Mediastinal only	No	187 (79.9%)	91 (77.8%)	96 (82.1%)	0.514
	Yes	47 (20.1%)	26 (22.2%)	21 (17.9%)	
Abdominal only	No	229 (97.9%)	115 (98.3%)	114 (97.4%)	1.000
	Yes	5 (2.1%)	2 (1.7%)	3 (2.6%)	
Multiple local	No	211 (90.2%)	102 (87.2%)	109 (93.2%)	0.188
	Yes	23 (9.8%)	15 (12.8%)	8 (6.8%)	
Recurrence treatment	No	62 (26.5%)	34 (29.1%)	28 (23.9%)	0.459
	Yes	172 (73.5%)	83 (70.9%)	89 (76.1%)	
	RT	18 (7.7%)	9 (7.7%)	9 (7.7%)	
	CT	68 (29.1%)	34 (29.1%)	34 (29.1%)	
	Surgery	9 (3.8%)	4 (3.4%)	5 (4.3%)	
	CRT	62 (26.5%)	27 (23.1%)	35 (29.9%)	
	TI	15 (6.4%)	9 (7.7%)	6 (5.1%)	

AJCC, American Joint Committee on Cancer; BMI, body mass index; LND, lymph node dissection; LNR, lymph node metastasis ratio; RT, radiotherapy; CT, chemotherapy; CRT, chemoradiotherapy; TI, targeted therapy and/or immunotherapy.

### Independent Prognostic Factors for PPS

**Figure 1:**
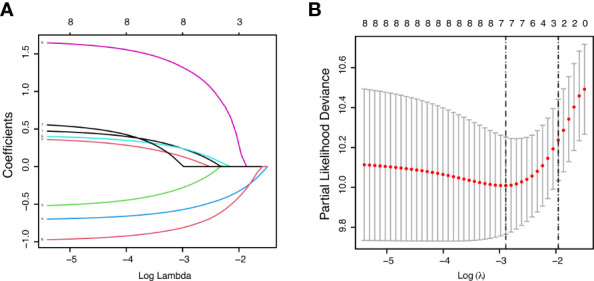
Feature selection using the least absolute shrinkage and selection operator (LASSO) Cox regression model. LASSO coefficient profiles of variables against the log lambda sequence for PPS **(A)** and tuning parameter (λ) selection in the LASSO model for PPS **(B)**.

In order to account for potential prognostic factors for PPS, the univariate Cox regression analysis was carried out based on the variables involving clinicopathological and recurrent characteristics, all of which met the proportional hazard assumption. All these incorporated factors were analyzed as categorical variables. Our results indicated that age, primary tumor location, BMI, number of LND, recurrence pattern, bone metastasis, pleura metastasis, and recurrence treatment were closely associated with the PPS (**Table 2**, all *P* < 0.05). LNR (HR =1.446, *P* = 0.056) and time to recurrence (HR =1.667, *P* = 0.053) also exhibited the relevance to PPS, however, without statistically significant difference. Remarkably, PPS was not significantly affected by the primary tumor features including the tumor size, degree of tumor differentiation, T-stage, and N-stage. In addition, we analyzed the lymph node metastasis at each station after surgery, but there was no significant correlation with PPS (**Supplementary Table 2**). LASSO analysis selected seven key prognostic indicators: age, primary tumor location, BMI, number of LND, recurrence pattern, bone metastasis, and recurrence treatment (**Figure 1**). Further multivariate Cox analysis revealed that age (HR =1.612, *P* = 0.045), BMI (HR =0.579, *P* = 0.026), number of LND (HR =0.489, *P* < 0.001), recurrence pattern (HR = 1.564, *P* = 0.032), bone metastasis (HR =5.170, *P* < 0.001), and recurrence treatment (HR =0.364, *P* < 0.001) were significant and independent indicators for predicting PPS in ESCC patients (**Table 2**).

**Table 2:** Independent prognostic factors for PPS.

Characteristics	Levels	n (%)	PPS					
			Univariate analysis			Multivariate analysis		
			HR	95% CI	*P*-value	HR	95% CI	*P*-value
Age	≤65 years	202 (86.3%)	1.723	1.088-2.731	0.020	1.612	1.010-2.573	0.045
	>65 years	32 (13.7%)						
Gender	Male	189 (80.8%)	1.142	0.730-1.786	0.561			
	Female	45 (19.2%)						
Tumor location	Upper third	43 (18.4%)	1.730	1.003-2.985	0.049	1.489	0.850-2.610	0.164
	Middle or lower third	191 (81.6%)						
Hypertension	No	213 (91.0%)	1.202	0.644-2.245	0.564			
	Yes	21 (9.0%)						
Diabetes	No	227 (97.0%)	0.642	0.158-2.605	0.536			
	Yes	7 (3.0%)						
Smoking	No	88 (37.6%)	1.042	0.710-1.528	0.835			
	Yes	146 (62.4%)						
Drinking	No	149 (63.7%)	1.153	0.781-1.703	0.473			
	Yes	85 (36.3%)						
Family tumor history	No	192 (82.1%)	1.079	0.658-1.770	0.763			
	Yes	42 (17.9%)						
Diet	Normal	88 (37.6%)	1.052	0.780-1.418	0.741			
	Semi-liquid	126 (53.8%)						
	Fluids	20 (8.5%)						
Weight loss	≤2.5 kg	176 (75.2%)	0.876	0.544-1.411	0.586			
	>2.5 kg	58 (24.8%)						
BMI	≤18.8 kg/m^2^	40 (17.1%)	0.548	0.346-0.869	0.011	0.579	0.358-0.936	0.026
	>18.8 kg/m^2^	194 (82.9%)						
Diagnostic date	Before 2010	94 (40.2%)	0.827	0.567-1.207	0.326			
	After 2010	140 (59.8%)						
Thoracic duct ligation	No	116 (49.6%)	1.019	0.701-1.482	0.922			
	Yes	118 (50.4%)						
Anastomotic leakage	No	199 (85.0%)	1.048	0.597-1.840	0.869			
	Yes	35 (15.0%)						
Differentiation	Well	49 (20.9%)	0.951	0.718-1.260	0.729			
	Moderately	130 (55.6%)						
	Poorly	55 (23.5%)						
T stage	T1b	13 (5.6%)	1.038	0.745-1.447	0.824			
	T2	39 (16.7%)						
	T3	178 (76.1%)						
	T4a	2 (0.9%)						
	T4b	2 (0.9%)						
N stage	N0	75 (32.1%)	0.978	0.792-1.207	0.833			
	N1	88 (37.6%)						
	N2	52 (22.2%)						
	N3	19 (8.1%)						
AJCC stage	IB	8 (3.4%)	0.992	0.867-1.136	0.910			
	IIA	31 (13.2%)						
	IIB	42 (17.9%)						
	IIIA	15 (6.4%)						
	IIIB	117 (50.0%)						
	IVA	21 (9.0%)						
Tumor size	≤2.0 cm	32 (13.7%)	1.237	0.693-2.207	0.472			
	>2.0 cm	202 (86.3%)						
Number of LND	≤10	28 (12.0%)	0.493	0.384-0.634	<0.001	0.489	0.381-0.626	<0.001
	>10, ≤18	58 (24.8%)						
	>18	148 (63.2%)						
LNR	≤0.07	131 (56.0%)	1.446	0.991-2.108	0.056			
	>0.07	103 (44.0%)						
Treatment after surgery	No	164 (70.1%)	0.907	0.702-1.171	0.452			
	CT	53 (22.6%)						
	RT	6 (2.6%)						
	CRT	11 (4.7%)						
Time to recurrence	≤6.2 months	40 (17.1%)	1.667	0.993-2.801	0.053			
	>6.2 months	194 (82.9%)						
Recurrence pattern	Local-regional	143 (61.1%)	1.726	1.181-2.522	0.005	1.564	1.040-2.352	0.032
	Distant metastasis	91 (38.9%)						
Lung only	No	215 (91.9%)	0.977	0.494-1.935	0.948			
	Yes	19 (8.1%)						
Liver only	No	222 (94.9%)	0.957	0.420-2.181	0.917			
	Yes	12 (5.1%)						
Bone only	No	225 (96.2%)	7.556	3.540-16.130	<0.001	5.170	2.307-11.585	<0.001
	Yes	9 (3.8%)						
Pleura only	No	230 (98.3%)	3.767	1.182-12.012	0.025			
	Yes	4 (1.7%)						
Other distant only	No	224 (95.7%)	1.630	0.713-3.725	0.247			
	Yes	10 (4.3%)						
Multiple distant	No	225 (96.2%)	1.144	0.421-3.108	0.792			
	Yes	9 (3.8%)						
Local Distant	No	206 (88.0%)	1.280	0.729-2.248	0.391			
	Yes	28 (12.0%)						
Anastomotic only	No	223 (95.3%)	0.371	0.091-1.505	0.165			
	Yes	11 (4.7%)						
Cervical only	No	177 (75.6%)	0.809	0.521-1.258	0.347			
	Yes	57 (24.4%)						
Mediastinal only	No	187 (79.9%)	0.692	0.422-1.136	0.146			
	Yes	47 (20.1%)						
Abdominal only	No	229 (97.9%)	0.592	0.083-4.251	0.603			
	Yes	5 (2.1%)						
Multiple local	No	211 (90.2%)	1.082	0.592-1.978	0.797			
	Yes	23 (9.8%)						
Recurrence treatment	No	62 (26.5%)	0.363	0.244-0.540	<0.001	0.364	0.241-0.551	<0.001
	Yes	172 (73.5%)						
	RT	18 (7.7%)						
	CT	68 (29.1%)						
	Surgery	9 (3.8%)						
	CRT	62 (26.5%)						
	TI	15 (6.4%)						

PPS, post-progression survival; HR, hazard ratio; CI, confidence interval; AJCC, American Joint Committee on Cancer; BMI, body mass index; LND, lymph node dissection; LNR, lymph node metastasis ratio; RT, radiotherapy; CT, chemotherapy; CRT, chemoradiotherapy; TI, targeted therapy and/or immunotherapy.

### Construction and Validation of a Prognostic Nomogram for PPS

**Figure 2:**
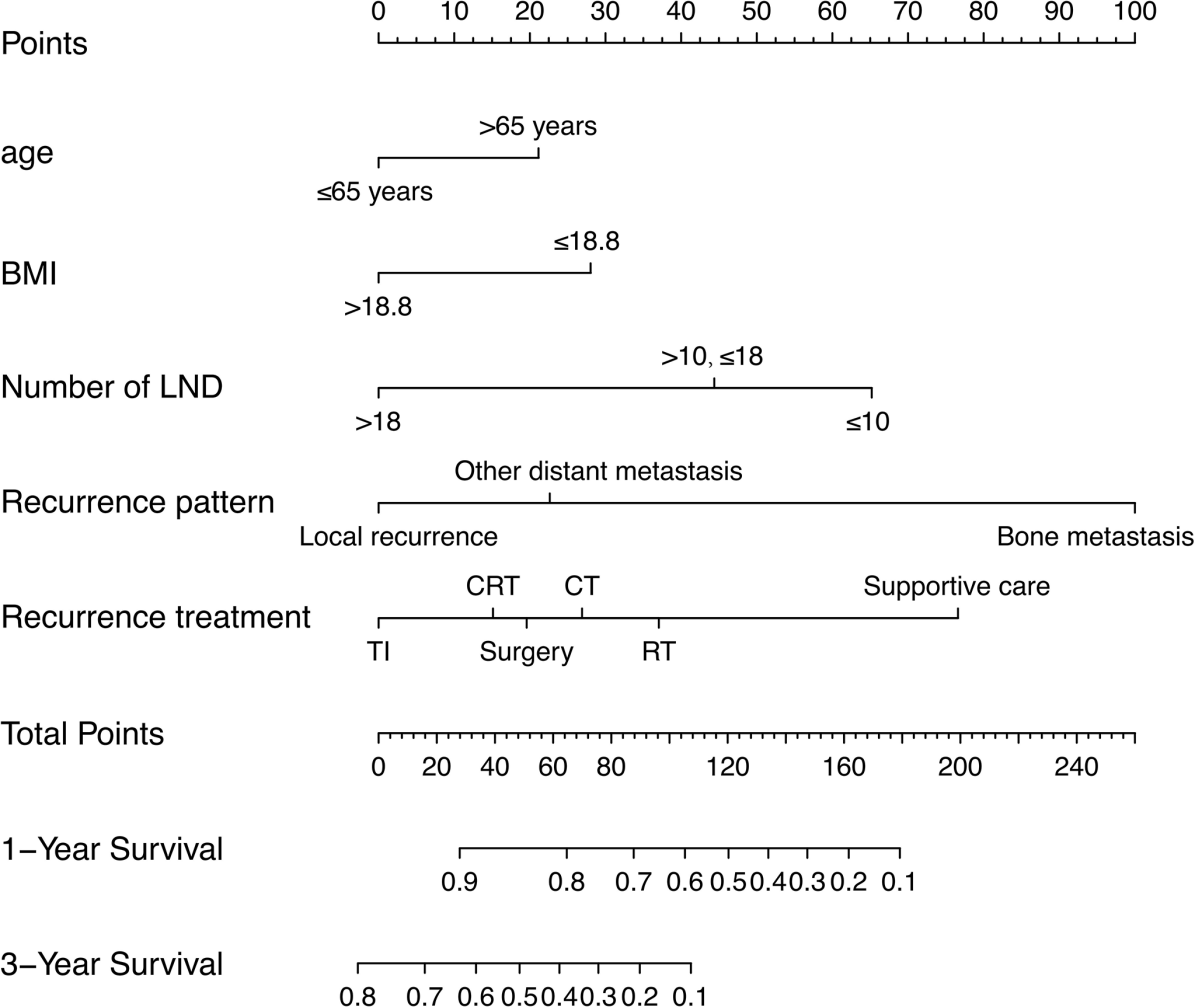
A prognostic nomogram for estimating the 1- and 3-year post-progression survival rates in the ESCC patients with recurrence after surgery.

**Figure 3:**
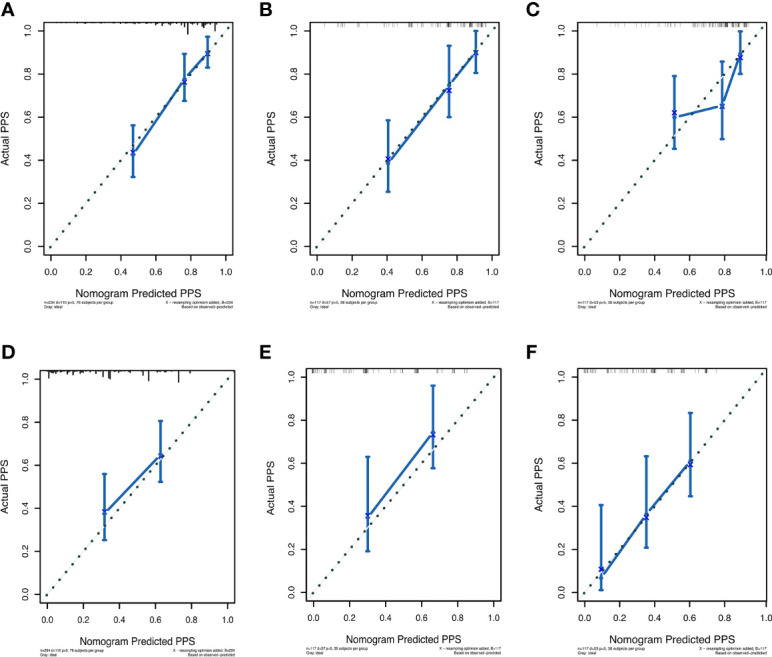
The calibration curves for predicting post-progression survival of ESCC patients at 1- and 3-year in the training cohort **(A, D)**, validation cohort 1 **(B, E)**, and validation cohort 2 **(C, F)**, respectively.

**Figure 4:**
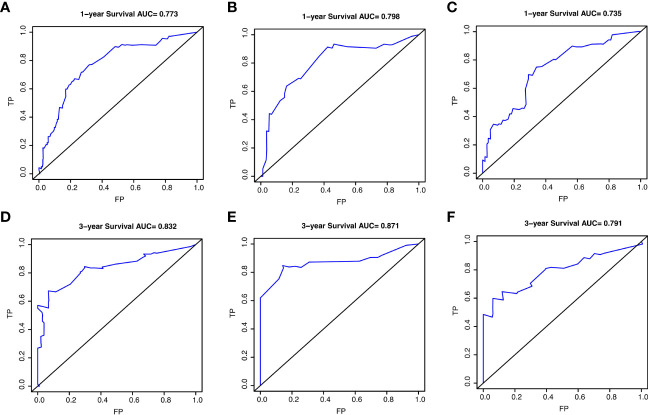
The receiver operating characteristic (ROC) curves for predicting post-progression survival of ESCC patients at 1- and 3-year in the training cohort **(A, D)**, validation cohort 1 **(B, E)**, and validation cohort 2 **(C, F)**, respectively.

**Figure 5:**
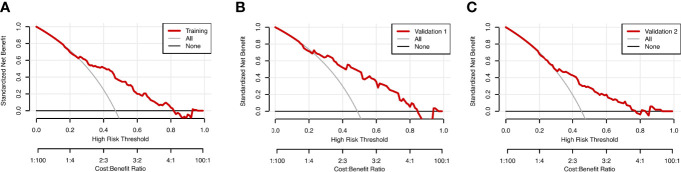
Decision curve analysis (DCA) for the nomogram model in the training cohort **(A)**, validation cohort 1 **(B)**, and validation cohort 2 **(C)** for PPS. The red lines represent the DCA of the nomogram.

The prognostic nomogram integrating above independent prognostic factors was established to predict 1- and 3-year PPS rates in 234 ESCC patients with recurrence after surgery (**Figure 2**). Importantly, recurrence pattern had the greatest effect on the prognosis of PPS, followed by the recurrence treatment, number of LND, BMI, and age. We next aimed to assess the efficacy of the nomogram. Calibration curves showed that the nomogram-predicted probability of 1- and 3-year PPS was highly consistent with the actual outcome in both training and validation cohorts (**Figure 3**). C-indexes of the nomogram in the training and two validation cohorts were 0.756 (0.733-0.779), 0.817 (0.791-0.843), and 0.730 (0.693-0.767), respectively (**Table 3**). C-index was 0.733 in the bootstrapping validation (Bootstrap = 1000). Furthermore, time-dependent ROC curves were shown in **Figure 4** and the 1- and 3-year AUC values in the training cohort were 0.773 and 0.832, demonstrating the satisfied predictive performance of the nomogram. Similar results (0.798 and 0.735 for 1-year AUC; 0.871 and 0.791 for 3-year AUC) were observed in two validation cohorts, which further confirmed the good reliability of the nomogram. Decision curve analysis displayed the clinical utility of the nomogram (**Figure 5**).

**Table 3:** The C-index and AUC values in the training and validation cohorts.

Cohort	PPS		
	C-index	AUC	
		1-year	3-year
Training cohort	0.756 (0.733-0.779)	0.773	0.832
Validation cohort 1	0.817 (0.791-0.843)	0.798	0.871
Validation cohort 2	0.730 (0.693-0.767)	0.735	0.791

PPS, post-progression survival; C-index, concordance index; AUC, area under receiver operating characteristic curve.

**Figure 6:**
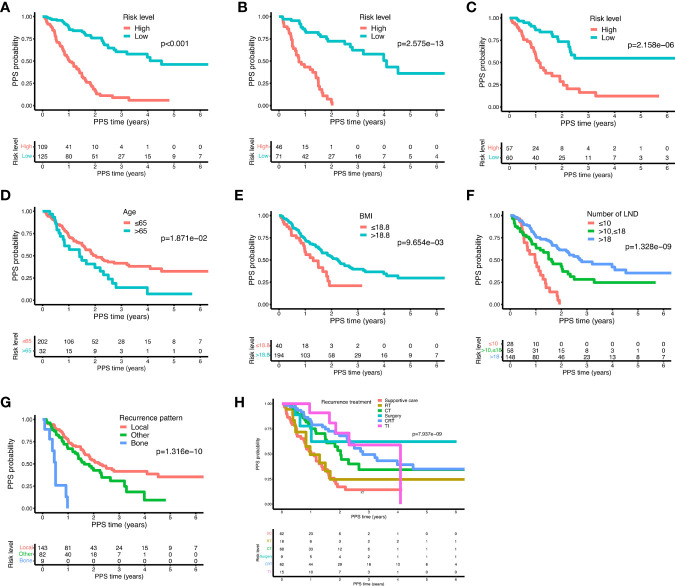
The Kaplan-Meier curves for the risk subgroups of ESCC patients. Patients were stratified by the prognostic score of the nomogram in the training cohort **(A)**, validation cohort 1 **(B)**, and validation cohort 2 **(C)**. Stratification of patients by significant characteristics after multivariate analysis in the training cohort: age **(D)**, BMI **(E)**, number of LND **(F)**, recurrence pattern **(G)**, and recurrence treatment **(H)**.

Based on the nomogram, each individual was assigned a total risk score and was then classified into the low- and high-risk subgroup according to the median value. As shown by the Kaplan-Meier curves, high-risk patients exhibited indeed significantly worse prognosis compared to patients in the low-risk group, which was subsequently demonstrated in two validation cohorts as well (**Figures 6A–C**, all *P* < 0.001). In the training cohort, age > 65 years, BMI ≤ 18.8 kg/m^2^, less number of LND, and distant metastasis subgroups presented significantly poorer PPS outcomes as compared to age ≤ 65 years, BMI > 18.8 kg/m^2^, more number of LND, and local-regional recurrence (**Figures 6D–G**, all *P* < 0.05), which was consistent with the result of the nomogram. Moreover, we found that among various treatments after recurrence, patients receiving targeted therapy and/or immunotherapy exhibited the best prognosis, followed by chemoradiotherapy, surgery, chemotherapy alone, radiotherapy alone, and supportive care (**Figure 6H**, *P* < 0.05).

Together, these data indicate that our nomogram provides an accurate and reliable method for predicting the PPS.

### Progression Pattern and PPS

**Figure 7:**
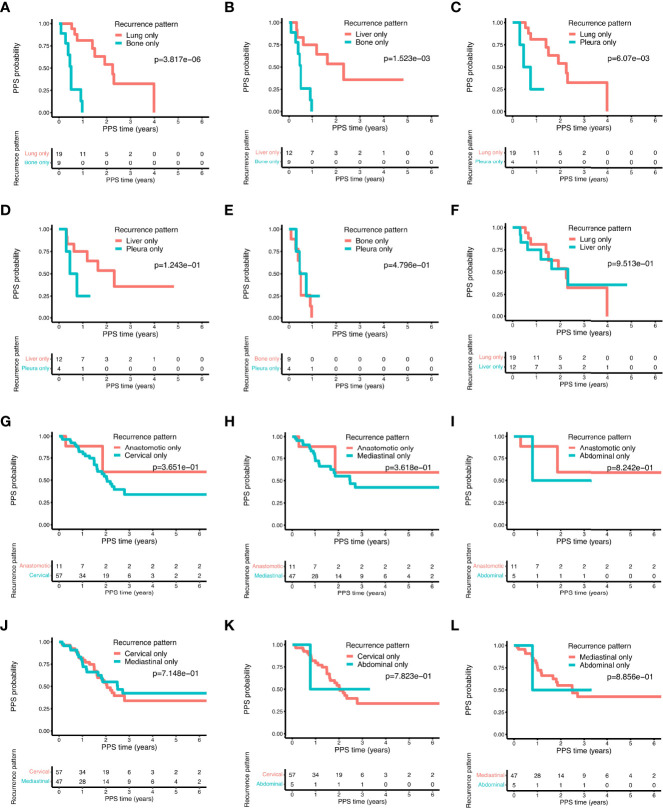
The comparison of post-progression survival of ESCC patients with different recurrence patterns in the training cohort. Comparisons of the post-progression survival based on the following distant recurrence patterns: bone only vs. lung only **(A)**; bone only vs. liver only **(B)**; lung only vs. pleura only **(C)**; liver only vs. pleura only **(D)**; bone only vs. pleura only **(E)**; liver only vs. lung only **(F)**. Comparisons of the post-progression survival based on the following local-regional recurrence patterns: anastomotic only vs. cervical only **(G)**; anastomotic only vs. mediastinal only **(H)**; abdominal only vs. anastomotic only **(I)**; cervical only vs. mediastinal only **(J)**; abdominal only vs. cervical only **(K)**; abdominal only vs. mediastinal only **(L)**.

Next, we analyzed whether the different progression patterns had distinct influences on the PPS. For isolated distant metastasis, bone only metastasis displayed a significantly worse prognosis as compared to lung only and liver only metastasis (**Figures 7A, B**, all *P* < 0.01). Pleura only metastasis had similar results as bone only metastasis showing a poorer survival outcome than lung only metastasis (**Figure 7C**, *P* < 0.01) and liver only metastasis (**Figure 7D**, *P* = 0.12). No statistically significant difference emerged between bone only and pleura only metastasis (**Figure 7E**). Furthermore, patients in lung only metastasis and liver only metastasis subgroups had similar PPS probability (**Figure 7F**). For local recurrence, no significant difference of PPS was observed in terms of different isolated recurrent sites, including the anastomotic stoma and the lymph nodes in the mediastinal, abdominal, and cervical areas (**Figures 7G–L**).

## Discussion

Recurrence was often encountered after radical resection of ESCC and greatly affected the prognosis of patients (23). Unlike the effect of predicting OS and PFS (7, 8), we found that TNM staging of primary tumor was not significantly associated with PPS in univariate analysis, suggesting that TNM staging was not effective in predicting PPS in ESCC patients. Similar to our conclusions, many studies on PPS of other cancer types suggested that the TNM staging system was less useful to predict the PPS prognosis (24, 25). Therefore, it is of practical significance to establish a nomogram for these patients. In this study, we explored the prognostic indicators for PPS based on clinicopathological characteristics and recurrent information of ESCC patients, after which a novel nomogram was established for the first time to predict the PPS of ESCC patients with recurrence after radical surgery. We demonstrated that the nomogram integrating age, BMI, the number of LND, recurrence pattern, and recurrence treatment was an accurate and reliable predictive approach to PPS in ESCC patients. Compared to previous PPS-related univariate/multivariate analysis (5, 6, 11, 12), our nomogram could predict PPS with the quantitative score.

Age was demonstrated predictive of OS and PFS of ESCC patients (26–28). Elderly patients were reported less benefit from intensive anti-cancer therapy (29). Butter *et al.* showed that older age was a risk factor for post recurrence survival of EC patients who underwent potentially curative esophagectomy (12). In line with this, advanced age was an unfavorable prognosis indicator of PPS in our study as well, especially for patients over 65 years of age, who were accompanied by an aging immune system (30), the poor functional reserve of multiple organ systems, and increased susceptibility to stimulation (31). With stratified analysis, Sugawara *et al.* revealed that elderly ESCC patients with low BMI had significantly poorer OS than patients with high BMI (32). Remarkably, EC patients with the recurrence were characterized by a lower BMI as compared to patients without the recurrence (33). Beyond that, low BMI was also found to be associated with worse PPS of ESCC patients in our work. BMI may affect prognosis by reflecting the nutritional status of ESCC patients (34). These results further highlight the importance of BMI in the prognosis of ESCC patients.

LND is known as a significant prognosis indicator of EC patients, however, the optimal number of resected lymph nodes remains controversial (35, 36). Based on the Surveillance Epidemiology and End Results database, it has been shown that with the number of LND increasing, the OS and cancer-specific survival were significantly increased in 4882 EC patients and patients receiving 30 or more LND had the lowest mortality rate when compared to other groups (37). National Comprehensive Cancer Network guidelines (Version 2.2022) recommended that at least 15 lymph nodes should be dissected for patients with EC, whether they received preoperative chemoradiotherapy or not. From the point of PPS, we found ESCC patients accepting more than 18 LND exhibited better clinical outcomes than patients in other groups, providing novel evidence of the contribution of LND and its exact quantity to the prognosis of ESCC.

Several retrospective studies confirmed the importance of salvage treatment for EC patients with recurrence. In the multivariate analysis of prognostic factors in 190 ESCC patients with recurrence after esophagectomy, Su *et al.* showed that treatment after recurrence was an independent prognostic factor for survival (5). Rodríguez-Camacho *et al.* also found that EC patients who received chemotherapy, radiotherapy, or a combination of both displayed a higher post-recurrence survival rate than palliative care (6.5 months vs. 2.9 months). However, the results were not statistically significant due to the limited sample size (33). Furthermore, Ni *et al.* reported that compared with those only treated with radiotherapy, the median PPS time of ESCC patients increased from 16.2 to 23.2 months after receiving chemoradiotherapy (11). Zhang *et al.* and Ni *et al.* put forward the specific salvage radiation dose and suggested that ≥60 Gy could effectively improve the prognosis of recurrent EC patients (11, 38). In the present study, we proved the effectiveness of salvage treatment for ESCC patients, and the targeted therapy and/or immunotherapy was found to be the optimal treatment for ESCC patients after recurrence.

The recurrence pattern was also part of the nomogram. Previously, it was demonstrated that local-regional recurrence and distant metastasis resulted in significantly different survival outcomes in ESCC after radical resection, and distant metastasis indicated poorer PPS (5), which was consistent with our results. In addition, we also analyzed features of subgroups of distant metastasis and local-regional recurrence. Among isolated distant metastases, lung only metastasis was the most common type, followed by liver only metastasis and bone only metastasis, which was identical with previous reports (5). Among them, bone only metastasis showed the greatest impact on PPS, which was worse than lung and liver metastasis. Indeed, various studies have demonstrated that bone metastasis in EC led to poorer PPS, as compared to liver or lung metastasis (39, 40). Beyond that, Zhang *et al*. reported that bone metastasis was particularly likely to happen to male EC patients in their study (39). For the local-regional recurrence, recurrences at various local-regional sites did not show different effects on PPS but were generally better than distant metastasis.

This study has important guiding value for clinicians, who can use these findings to assess the PPS among patients with ESCC following radical resection and provide patients with individual recommendations and measures. For instance, for patients with high-risk factors, such as older age, intensive follow-up should be recommended. Patients need to be reminded to pay attention to BMI and strengthened their postoperative nutrition. Furthermore, it also reminds the surgeon that an appropriate number of lymph nodes should be removed during the operation. More vigilance should be raised when distant metastasis occurs in postoperative patients, especially bone metastasis. Active treatment is necessary and can significantly improve prognosis. Therefore, the results of this study can be used to predict survival after the progression of ESCC and are potential of benefit to the intervention of decision-making.

However, several limitations need to be pointed out in this study. First, the sample size was still relatively small. We expect to enroll more patients and even verify in prospective studies in the future. In addition, although the prognostic model showed good fitness after validation, we should recognize that our validation cohorts were derived from the training cohort, so it would be better to further validate our nomogram in more independent cohorts.

In conclusion, we have developed a nomogram to predict PPS in ESCC patients with recurrence after radical surgery. Validation by training and validation cohorts showed that the nomogram had a strong predictive ability for PPS. In addition, we also compared the PPS of different recurrence patterns. The establishment of this nomogram could provide new insights into the individualized ESCC patients with recurrence.

## Data Availability Statement

The raw data supporting the conclusions of this article will be made available by the authors, without undue reservation.

## Ethics Statement

The Institutional Review Board of Sun Yat-Sen University Cancer Center approved this study. The patients/participants provided their written informed consent to participate in this study. Written informed consent was obtained from the individual(s) for the publication of any potentially identifiable images or data included in this article.

## Author Contributions

QL, JF, and JW conceptualized and designed the study. QL, CL, YC, JC, KW, HY, and JW collected and assembled the data. QL, CL, and YC analyzed and interpreted the data. All authors have contributed to the article and approved the submitted version.

## Funding

This work was supported by grant from the China Postdoctoral Science Foundation (No. 2021M693655).

## Conflict of Interest

The authors declare that the research was conducted in the absence of any commercial or financial relationships that could be construed as a potential conflict of interest.

## Publisher’s Note

All claims expressed in this article are solely those of the authors and do not necessarily represent those of their affiliated organizations, or those of the publisher, the editors and the reviewers. Any product that may be evaluated in this article, or claim that may be made by its manufacturer, is not guaranteed or endorsed by the publisher.
