# Supplementary material for: A Nomogram Model to Predict Post-Progression Survival in Esophageal Squamous Cell Carcinoma Patients With Recurrence After Radical Resection
Source: Front Oncol. 2022 Jul 7;12:925685. doi: 10.3389/fonc.2022.925685 (PMC9300830; doi:10.3389/fonc.2022.925685)
Supplement: Supplementary file 1 [file Table_1.docx]

Supplementary Table 1. References of R-packages.

| R-package | Reference |
| --- | --- |
| survival | https://doi.org/10.1002/sim.956 |
| survminer | https://CRAN.R-project.org/package=survminer |
| rms | https://CRAN.R-project.org/package=rms |
| glmnet | https://www.jstatsoft.org/v33/i01/ |
