# Supplementary material for: A Nomogram Model to Predict Post-Progression Survival in Esophageal Squamous Cell Carcinoma Patients With Recurrence After Radical Resection
Source: Front Oncol. 2022 Jul 7;12:925685. doi: 10.3389/fonc.2022.925685 (PMC9300830; doi:10.3389/fonc.2022.925685)
Supplement: Supplementary file 2 [file Table_2.docx]

Supplementary Table 2 | Lymph node metastasis in the postoperative pathology as prognostic factors for PPS.

| **Characteristics** | **Levels** | **n (%)** | **PPS** | | |
| --- | --- | --- | --- | --- | --- |
|  |  |  | **Univariate analysis** | | |
|  |  |  | **HR** | **95% CI** | ***P*-value** |
| Right recurrent laryngeal nerve LNM | No | 173 (73.9%) | 1.022 | 0.671-1.556 | 0.920 |
|  | Yes | 61 (26.1%) |  |  |  |
| Left recurrent laryngeal nerve LNM | No | 202 (86.3%) | 0.728 | 0.380-1.396 | 0.340 |
|  | Yes | 32 (13.7%) |  |  |  |
| 2, 4 LNM | No | 232 (99.1%) | 1.747 | 0.429-7.107 | 0.436 |
|  | Yes | 2 (0.9%) |  |  |  |
| 7 LNM | No | 200 (85.5%) | 1.005 | 0.591-1.711 | 0.985 |
|  | Yes | 34 (14.5%) |  |  |  |
| 8 LNM | No | 162 (69.2%) | 0.808 | 0.533-1.224 | 0.314 |
|  | Yes | 72 (30.8%) |  |  |  |
| 9 LNM | No | 233 (99.6%) | 1.286 | 0.179-9.245 | 0.802 |
|  | Yes | 1 (0.4%) |  |  |  |
| Diaphragm LNM | No | 229 (97.9%) | <0.001 | 0.000-Inf | 0.994 |
|  | Yes | 5 (2.1%) |  |  |  |
| Cardia LNM | No | 203 (86.8%) | 0.964 | 0.539-1.723 | 0.901 |
|  | Yes | 31 (13.2%) |  |  |  |
| Left gastric artery LNM | No | 188 (80.3%) | 1.049 | 0.651-1.688 | 0.845 |
|  | Yes | 46 (19.7%) |  |  |  |
| Common hepatic artery LNM | No | 232 (99.1%) | 0.915 | 0.127-6.577 | 0.930 |
|  | Yes | 2 (0.9%) |  |  |  |
| Celiac artery LNM | No | 230 (98.3%) | 0.994 | 0.138-7.162 | 0.995 |
|  | Yes | 4 (1.7%) |  |  |  |
| Splenic artery LNM | No | 233 (99.6%) | <0.001 | 0.000-Inf | 0.996 |
|  | Yes | 1 (0.4%) |  |  |  |
| Lesser curvature LNM | No | 223 (95.3%) | 0.929 | 0.378-2.279 | 0.871 |
|  | Yes | 11 (4.7%) |  |  |  |
| Greater curvature LNM | No | 233 (99.6%) | <0.001 | 0.000-Inf | 0.995 |
|  | Yes | 1 (0.4%) |  |  |  |

*PPS, post-progression survival; HR, hazard ratio; CI, confidence interval; LNM: lymph node metastasis; Inf: infinity. Diaphragm LNM includes superior diaphragm LNM, inferior diaphragm LNM, and esophageal hiatus LNM.*
